# CD9 Expression by Human Granulosa Cells and Platelets as a Predictor of Fertilization Success during IVF

**DOI:** 10.1155/2010/192461

**Published:** 2010-09-01

**Authors:** Carolyn R. Jaslow, Kyle S. Patterson, Shila Cholera, Lisa K. Jennings, Raymond W. Ke, William H. Kutteh

**Affiliations:** ^1^Department of Biology, Rhodes College, 2000 North Parkway, Memphis, TN 38112, USA; ^2^Department of Obstetrics and Gynecology, University of Tennessee Health Science Center, Memphis, TN 38152, USA; ^3^Department of Internal Medicine, The Vascular Biology Center of Excellence, University of Tennessee Health Science Center, Memphis, TN 38152, USA; ^4^Fertility Associates of Memphis, 80 Humphreys Center, Suite 307, Memphis, TN 38120, USA

## Abstract

*Objective*. To determine whether CD9 expression on human granulosa cells (GCs) and platelets could predict the success of conventional fertilization of human oocytes during in vitro fertilization (IVF). *Methods*. Thirty women undergoing IVF for nonmale factor infertility participated. Platelets from venous blood and GCs separated from retrieved oocytes were prepared for immunofluorescence. Flow cytometry quantified the percent of GCs expressing CD9, and CD9 surface density on GCs and platelets. Fertilization rate was determined for the total number of oocytes, and the number of mature oocytes per patient. Correlations tested for significant relationships (*P* < .05) between fertilization rates and CD9 expression. *Results*. CD9 surface density on human GCs is inversely correlated with fertilization rate of oocytes (*P* = .04), but the relationship was weak. *Conclusion*. More studies are needed to determine if CD9 expression on GCs would be useful for predicting conventional fertilization success during IVF.

## 1. Introduction

Infertility, defined as the inability to conceive for at least one year, is an emotionally devastating problem that affects about 7.4% of reproductive-age married women in the United States [1]. At least 85% of the time, hormonal therapies and surgery can resolve the problems with conception [2], but for some couples, Assisted Reproductive Technology (ART) procedures, such as in vitro fertilization (IVF), are the best chance for reproductive success. During conventional IVF, fertilization involves the introduction of sperm into microdroplets containing secondary oocytes. Success depends on the ability of the sperm to approach the oocyte, penetrate though the outer cumulus and zona layers, and then bind and fuse with the oocyte's plasma membrane. In the early 1990s, however, intracytoplasmic sperm injection (ICSI) was introduced as an alternative method of assisted fertilization [3]. During ICSI, a selected sperm is inserted directly into the oocyte's cytoplasm, so this technique bypasses the usual processes of sperm-oocyte penetration, binding, and fusion.

Since its introduction, ICSI has become increasingly common, primarily because of its undisputed success for couples with male-factor infertility [4]. More recently, some practitioners have argued that ICSI should be used for all IVF procedures [5, 6], and the use of ICSI in cases unrelated to male-factor infertility has increased greatly at ART facilities worldwide [7–9]. Such general use of ICSI, however, raises concerns because of its greater costs, not only the additional financial burden to the patient, but also the lab time spent performing sperm microinjections on numerous retrieved oocytes. Additionally, ICSI might increase the risk of transmitting chromosomal anomalies or imprinting disorders (see reviews [10–12]), although it is not clear whether these risks are due to the procedure or to the factors causing male infertility [8].

At present, there are no morphological or physiological features of oocytes that can predict whether conventional fertilization will be successful, or whether there is a need for ICSI. Given the risks and costs associated with ICSI, it would be helpful to have an independent marker that could predict, a priori, the likelihood of successful fertilization with conventional IVF for couples with nonmale factor infertility. The sperm and oocyte both express numerous cell surface proteins involved in the fertilization process. In particular, the fusion of the sperm and egg membranes is a crucial step that has been well studied, but is not completely understood [13]. On the oocyte, one protein necessary for gamete fusion is the tetraspanin, CD9. Female mice lacking a functional CD9 gene are healthy and grow normally, but are infertile because their oocytes cannot fuse with sperm [14–16]. This function can be restored if CD9 deficient oocytes are injected with CD9 mRNA [17, 18].

Because oocyte CD9 is needed for gamete fusion, measuring the expression of CD9 in women could provide a useful marker for predicting conventional IVF fertilization success in couples with normal sperm parameters, but nonmale factor infertility. Potentially, high expression of CD9 could indicate a high probability of oocyte fertilization using conventional IVF whereas low expression might justify a need for ICSI instead, although such a relationship has never been measured. Ideally, oocytes from women undergoing IVF for nonmale factor infertility could be tested for the presence of CD9. Unfortunately, this procedure would use oocytes needed for fertilization, which have been collected after an expensive, time-consuming, and uncomfortable procedure. CD9, however, is also expressed on the granulosa cells surrounding the oocytes, and on circulating peripheral blood platelets [19, 20]. These cells can easily be collected, either before (platelets) or during oocyte retrieval, and their expression of CD9 could be measured in time to make a decision whether conventional fertilization would likely be successful for that IVF cycle. CD9 expression on granulosa cells and platelets has been linked with female reproductive function [20–22], however, no study has tested whether CD9 expression on granulosa cells and platelets correlates with CD9 expression on oocytes. The specific goal of this study, therefore, was to determine whether CD9 expression by granulosa cells and platelets collected the day of oocyte retrieval could predict the fertilization success for oocytes obtained during a conventional IVF cycle.

## 2. Method

### 2.1. Participants

This study was approved by the Institutional Review Board at the University of Tennessee Health Sciences Center and at Rhodes College, and all participants gave informed consent. The subjects were 30 women who were undergoing IVF for nonmale factor infertility; that is, their partners had normal sperm parameters. The mean age of the patients (+1 SE) was 32.5 years (+0.77), and a preliminary analysis showed no correlations (*P* > .05) between patient age and oocyte fertilization rate or CD9 expression. Patient ethnicity was 90% Caucasian, 5% Asian, and 5% African-American. Controlled ovarian stimulation was accomplished using pituitary suppression in the luteal phase with GnRH agonist (Luprolide acetate, Tap Pharmaceuticals, Lake Forest, IL). Recombinant FSH (Gonal F, EMD-Serono, Rockland, MD) or Follistim AQ (Schering-Plough, Roseland, NJ) was used to induce multiple follicle development. When at least three follicles with a mean diameter >18 mm were observed on ultrasound, 10,000 mIU hCG (Profasi or Ovidrel, EMD-Serono) was administered followed by oocyte retrieval 35 hours later.

### 2.2. Granulosa Cell and Platelet Collection and Preparation

Oocytes with surrounding granulosa cells (GCs) were retrieved from patients using an ultrasound-guided transvaginal puncture. Only follicles that were greater than 14 mm in diameter were aspirated so that the cohort of oocytes and GC from all patients would be comparable in maturation. Subsequently, the GCs were separated from the retrieved oocytes, and GCs from all follicles per patient were pooled together in modified human tubal fluid. After mechanical dispersion using a pipette, the GC solution was layered over Histopaque 1077 (Sigma) and centrifuged at 600 g for 20 minutes at room temperature. GCs from the interface were washed in Dulbecco's Modified Eagle Medium: Nutrient Mixture F-12 (DMEM) with 1% newborn calf serum (NCS) (Invitrogen) and centrifuged (600 g, 5 min) twice. The supernatant was discarded and the GCs were resuspended in DMEM +1% NCS to produce a cell concentration of 1 × 10^6^ viable cells/mL, based on trypan blue staining for viability. To obtain platelets from patients on the day of oocyte retrieval, a venous blood sample was collected in a 4.5 mL glass tube with 3.2% buffered sodium citrate (BD Diagnostics). From this whole blood sample, platelet-rich plasma (PRP) was isolated by differential centrifugation, and platelets were counted using a Coulter Counter. Platelet-poor plasma (PPP) was used to dilute the PRP to a concentration of 2.5 × 10^8^cells/mL.

### 2.3. Monoclonal Antibodies for Flow Cytometry

Mouse anti-human CD9 mAb7 [23], provided by Dr. Lisa K. Jennings, was conjugated with B-phycoerythrin (Invitrogen) to provide anti-CD9-PE antibodies. Mouse anti-human CD45, conjugated with fluorescein isothiocyanate (anti-CD45-FITC) (Santa Cruz), was used to mark leukocytes to distinguish them from the granulosa cells [24], and normal mouse IgG_1_-PE and IgG_1_-FITC (Santa Cruz) were used as negative isotype controls.

### 2.4. Staining Procedure

Eppendorf tubes containing 100 *μ*L aliquots of the GC solution for each patient (10^5^ viable cells/100 *μ*L) were incubated with the following combinations of conjugated antibodies: 5 *μ*L anti-CD9-PE and 5 *μ*L anti-CD45-FITC, or 5 *μ*L mouse IgG_1_-PE and 5 *μ*L mouse IgG_1_-FITC (isotype controls). Tubes containing 100 *μ*L aliquots of each platelet solution (2.5 × 10^7^ cells/100 *μ*L) were incubated with 8 *μ*L anti-CD9-PE and 2.5 *μ*L sequesol, or 8 *μ*L mouse IgG_1_-PE (isotype control). Additional aliquots of GCs and of platelets were left unstained as controls. All tubes were incubated in the dark at room temperature for 30 minutes. Subsequently, the GCs and platelets were centrifuged (800 g for 5 minutes) and washed three times with cold PBS before they were resuspended in 500 *μ*L of cold PBS for flow cytometry. For positive controls, Raji cells were stained and processed as described above. 

### 2.5. Flow Cytometric Analysis

**Figure 1:**
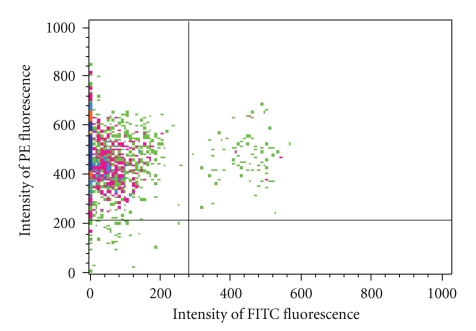
Flow cytometry separation of granulosa cells (GCs) from leukocytes based on intensity of staining with anti-CD45-FITC. Events showing CD45^+^ leukocytes are located in the upper right quadrant whereas those representing CD45^−^ GCs occur in the upper left. The bottom two quadrants contain a few cells that did not pick up the anti-CD9-PE antibody.

A FACSCalibur (Becton Dickinson) was used for flow cytometric analysis of GCs and platelets. Size gating of the GC sample was used to eliminate any debris and the few contaminating blood cells that were not the size of GCs (e.g., erythrocytes). During analysis of each sample, the cursor was placed so that <2% of the cells incubated with the isotype control antibodies (IgG_1_-PE and IgG_1_-FITC) appeared as positive staining events. Staining with anti-CD45-FITC allowed separation of the CD45^+^ leukocytes from the CD45^−^ GCs [24] (Figure 1). CD9 expression by GCs was quantified two ways: the percent of GCs expressing CD9-PE and the surface density of CD9. CD9 surface density was measured as the mean relative fluorescence intensity for all GCs (CD45^−^ cells) that stained positively for CD9-PE in each sample. For platelets, only surface density of CD9 was measured.

### 2.6. Oocyte Fertilization Rate

Fertilization rate was calculated as the percent of oocytes that progressed to the two pronuclei/two polar body stage (2PN/2PB) 16 hours after conventional fertilization, and again at 24 hours after fertilization. Fertilization rate was determined for both the total number of oocytes retrieved per patient, as well as for the number of mature oocytes. Oocyte maturity was assessed at the time of insemination based on cumulus expansion, and it was estimated that 85%–95% of the oocytes appeared mature. This was confirmed at 16 hours and 24 hours by microscopic examination, which identified 90% of the oocytes to be either fertilized, or at least in the metaphase II stage (had produced one polar body). Although all sampled follicles were at least 14 mm in diameter to help ensure similar oocyte maturity, the calculation of fertilization rate was done for mature oocytes, in addition to the rate for all oocytes, to eliminate oocytes that were obviously too immature for fertilization.

### 2.7. Statistical Analysis

Pearson correlation coefficients and Model II regression [25] were used to test for significant relationships (*P* < .05) between fertilization rates and CD9 expression. Fertilization and GC percentages were arcsine transformed to normalize the data prior to data analysis and graphing [25]. Figures show transformed data, but percentage values and means ± standard errors of the means (SEM) in the text represent untransformed data. This study was powered to detect a 15% difference in fertilization rate with *α* = 0.05 and *β* = 0.80 with 29 subjects.

## 3. Results

The frequency of GCs that expressed CD9 varied from 25% to 92%  of the GCs retrieved from each of the 30 patients, with a mean frequency of 64% ± 8.8 CD9^+^ GCs/all GCs. The mean intensity of CD9-PE fluorescence on the GCs was 345 ± 7.6. For platelets, the mean CD9-PE fluorescence intensity was 117 ± 6.6. 

The number of oocytes retrieved per patient ranged from 4 to 35, with a mean of 16.0 ± 1.4 oocytes per patient. Of these retrieved oocytes, the mean frequency of mature oocytes per patient was 90% (14.4 ± 1.3 oocytes). The mean number of fertilized oocytes was 10.0 ± 1.0 oocytes per patient. This yielded mean fertilization rates of 62% for all oocytes retrieved (range = 20%–80%), and 72% (range = 27%–100%) for all mature oocytes.

**Figure 2:**
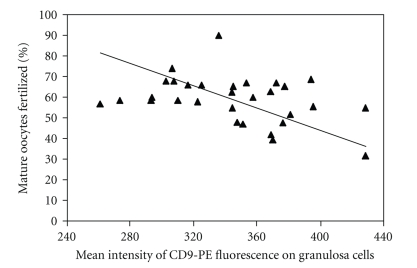
Greater surface density of CD9 on the GCs (measured as mean intensity of CD9-PE fluorescence) correlated with lower fertilization rates in mature oocytes (*r* = − 0.372, *P* = .04). See the text for the definition of mature oocytes. The Model II regression is *y* = − 0.27*x* + 152.8.

The rate of fertilization among mature oocytes showed a slight inverse correlation with greater CD9 surface density (i.e., CD9-PE fluorescence intensity) (*r* = −0.372, *P* = .04) (Figure 2). This relationship was relatively weak. The coefficient of determination, *R* ^2^, is only 0.14, indicating that the intensity of CD9 expression on the surface of the GCs only explained 14% of the variation in fertilization rate among the mature oocytes. No correlations were found between fertilization rate and the percent of GCs expressing CD9, or between fertilization rate and CD9 surface density on platelets (Table 1).

## 4. Discussion

Surface density of CD9 on pooled GCs was inversely correlated with fertilization frequency among mature oocytes. However, because it explained so little of the variance in oocyte fertilization rate, our data suggest it would not be a useful marker to predict whether conventional fertilization would be successful during IVF. Most likely, pooling of GCs from all >14 mm follicles in each patient reduced the sensitivity of the analysis compared to testing GCs from each follicle. In addition, pooling of GCs from all follicles did not allow analysis of GCs from only the follicles containing mature oocytes. We anticipated that this was a minor reduction in sensitivity because 90% of the follicles contained mature oocytes. Pooling was needed, however, to provide a sufficient supply of cells for flow cytometry, and it ensured that the analysis could be efficiently completed within the brief window of time between oocyte retrieval and fertilization.

The use of ICSI during IVF is clearly indicated for cases of oligoasthenoteratozoospermia (low sperm count with numerous slow-moving and abnormal sperm) as well as for those with either obstructive or nonobstructive azoospermia [10]. In addition, ICSI is indicated for couples undergoing preimplantation genetic diagnosis (PGD) because it reduces the risk that DNA extracted from the embryo would be contaminated by residual DNA from any sperm still surrounding the embryo following conventional fertilization [7, 10]. Yet more and more practitioners worldwide are using ICSI for patients without male-factory infertility or PGD [5–9]. This may be attributed, in part, to findings that ICSI generates higher fertilization rates compared to conventional IVF [26]. Given the additional costs and potential risks of the procedure [10–12], there remains a need for a marker to predict whether conventional fertilization would likely be successful in any given IVF cycle.

The results of this study indicate that CD9 expression on human GCs and platelets may not be able to fulfill the role of predicting conventional fertilization success during IVF, but other cell surface proteins on GC's could be tested to determine if any may be able to perform that function. Additionally, there are several genes expressed within the GCs that could be evaluated for this purpose because they have already been identified as markers for oocyte competence (i.e., the ability of oocytes to yield a successful pregnancy). These include genes induced during cumulus cell expansion (e.g., *HAS2, PTGS2, GREM1, PTX3*), genes involved in synthesis of progesterone (e.g., *FDX1, 3*β*HDS1*) or estrogen (*CYP19A1*), and genes induced by the LH surge (e.g., *STAR, COX2*) [27–31]. Expression of most of these GC genes was correlated with developmental success of oocytes following ICSI, consequently, these may not be useful markers to predict success of conventional fertilization. Of two studies that used oocytes that were fertilized conventionally, one evaluated competence based on whether the embryos reached the 8-cell stage and resulted in a successful pregnancy following transfer [29]. The five GC genes identified, including *FDX1, 3*β*HDS1*, and *CYP19A1*, may correlate with oocyte competence leading to fertilization, but there is also the possibility that these genes associate with successful early cleavage instead. The second study to use oocytes that had been fertilized conventionally compared GCs from follicles of oocytes that failed to fertilize with GC's associated with oocytes that reached the 8-cell stage successfully. Furthermore, the cells used were cumulus cells, the GCs immediately surrounding the oocytes. Their DNA microarray identified numerous cumulus cell genes with differential expression associated with oocyte fertilization [31]. One of these genes was *PTX3*, which is involved in proper structuring of the oocyte/cumulus complex and is important for female fertility [32]. Female knockout mice lacking the *PTX3* gene not only ovulate fewer oocytes, but their oocytes have lower fertilization rates [32]. Both *PTX3* and other GC genes identified in these studies could be further tested for their usefulness as markers for conventional fertilization success during IVF.

In contrast to oocyte CD9, which is necessary for fertilization [14–16], it appears that CD9 on GCs serves a different function that is more indirectly related to fertilization success. On GCs, CD9 associates with *α*_6_ *β*_1_, an integrin heterodimer that binds laminin, a component of the extracellular matrix surrounding the GCs [20]. Although Fujiwara et al. [33] did not detect laminin in the matrix between GCs of preovulatory follicles, they did find it bound to GCs collected during oocyte retrieval for IVF (i.e., at the time of ovulation). In humans, the interaction of laminin with *α*_6_ *β*_1_ on GCs inhibits the cells' maturation into granulosa-lutein cells that secrete progesterone [33]. Expression of both CD9 and *α*_6_ increases on GCs leading up to ovulation [20, 34–36], and the appearance of laminin in the matrix around the time of ovulation may be important for preventing premature luteinization of the GCs. At ovulation, however, laminin's inhibition of GC maturation must stop to permit formation of the corpus luteum and secretion of progesterone. Because laminin remains present in the GC matrix from ovulation up to four days afterwards [33], the mechanism to reduce its impact most likely lies in or on the GCs themselves.

To block the inhibitory effects of laminin at ovulation, it is possible that the GCs decrease expression of the *α*_6_ *β*_1_ integrin to which laminin binds. Although immunohistochemistry has shown that *α*_6_ expression increases on GCs leading up to ovulation, and persists on GCs during corpus luteum formation [20, 34–36], flow cytometry by Clavero et al. [21] demonstrated that GCs from mature follicles with metaphase II oocytes expressed significantly less *α*_6_ than GCs from immature follicles with only metaphase I oocytes. This downregulation of *α*_6_ at ovulation may be sufficient to mitigate laminin's suppression of GC maturation. It is also likely that GCs mature because intracellular signals reduce the affinity of *α*_6_ *β*_1_ to the available laminin [37, 38]. This loss of affinity by an integrin may occur through rapid changes in its extracellular domains [38, 39], or by changes in integrin position or clustering [40, 41]. CD9 promotes the clustering of *α*_6_ *β*_1 _ [42]. Like *α*_6_, CD9 expression on GCs increases though ovulation [20], but at ovulation there is less CD9 on GCs of mature follicles than immature ones [21]. This reduction in CD9 expression by GCs at ovulation should cause dispersal of the *α*_6_ *β*_1_ integrin from its clustered arrangement, and a concomitant loss of *α*_6_ *β*_1_ affinity for laminin. The decrease of laminin signaling would then permit the GCs' maturation into progesterone-secreting granulosa-lutein cells. Microarray analysis has shown that greater expression of GC genes promoting progesterone synthesis is significantly correlated with production of competent oocytes [29]. Perhaps the negative correlation we observed between CD9 expression in GCs and fertilization rate in metaphase II oocytes reflects some variation in the GC maturation and competence of oocytes among the patients. Our samples of GCs came from all of the follicles greater than 14 mm that produced oocytes during the retrieval process, so a patient with a greater proportion of mature follicles producing oocytes capable of conventional fertilization may have had somewhat lower expression of GC CD9 that a patient with fewer mature follicles.

Though CD9 on GCs is not directly involved in fertilization of the oocyte, it may affect fertilization indirectly though its role in follicle maturation. A mature follicle, usually defined by its large size, generally has a greater probability of containing an oocyte with a mature nucleus and cytoplasm. There is considerable communication between the GCs and an oocyte in a developing follicle, and gene expression in maturing GCs is closely associated with the production of competent oocytes [27, 29, 43, 44]. Platelets, however, are far removed from oocyte maturation and fertilization, so it is not surprising that platelet CD9 expression was completely unrelated to fertilization rates among oocytes. Platelets were included in this study because they express abundant CD9, they are very simple to obtain without discomfort or injury to a patient, and because data from a preliminary study suggested that platelet CD9 might be linked with fertilization rates [22]. In this prior investigation, platelets were sampled from study subjects at different times during their menstrual or IVF cycles. Because platelet function and platelet-derived microparticle activity are affected by both endogenous and exogenous reproductive steroid hormones [45–48], it is possible that prior indication of a link between platelets and fertilization may have been a byproduct of variation in the time of sampling among patients.

## 5. Conclusion

Measurement of CD9 expression on human granulosa cells and platelets may not be a useful indicator for predicting the success of conventional fertilization in couples undergoing IVF. A weak negative relationship between surface density of CD9 on granulosa cells and fertilization rate of mature oocytes may reflect a downregulation of CD9 that accompanies follicle maturation at ovulation. More studies are necessary to determine the role of CD9 on granulosa cells during follicle maturity, and to assess if CD9 expression on human granulosa cells could be used as a factor to predict the success of conventional fertilization during IVF. 

## Figures and Tables

**Table 1:** Correlations between CD9 expression on human granulosa cells (GCs) and platelets and fertilization rates in oocytes.

	Correlation with fertilization rate in all oocytes		Correlation with fertilization rate in mature oocytes^a^	
Measure of CD9 expression	Correlation coefficient	*P*-Value	Correlation coefficient	*P*-Value
Percent of GCs expressing CD9 (*n* = 30)	0.151	.43	0.135	.48
Surface density of CD9^b^ on GCs (*n* = 30)	−0.316	.09	−0.372	.04
Surface density of CD9^b^ on platelets (*n* = 26)	−0.141	.49	−0.029	.89

^a^See the text for the definition of mature oocytes.

^b^CD9 surface density was measured as the mean intensity of CD9-PE fluorescence during flow cytometry.
